# Evaluation of the Effectiveness and Safety of New Wound Coatings Based on Cod Collagen for Fast Healing of Burn Surfaces

**DOI:** 10.3390/polym17233215

**Published:** 2025-12-02

**Authors:** Anna Soloveva, Lyudmila Semenycheva, Victoria Rumyantseva, Yulia Kuznetsova, Veronika Prodaevich, Natalia Valetova, Petr Peretyagin, Natalia Didenko, Ksenia Belyaeva, Diana Fukina, Maria Vedunova, Evgeny Suleimanov

**Affiliations:** Lobachevsky State University of Nizhny Novgorod, pr. Gagarina 23, 603950 Nizhny Novgorod, Russia; sannag5@mail.ru (A.S.); vo.rumyantseva@mail.ru (V.R.); kyul@yandex.ru (Y.K.); prodaevitchnika@yandex.ru (V.P.); nata-bor-2005@mail.ru (N.V.); peretyaginpv@gmail.com (P.P.); natalika-nv@mail.ru (N.D.); skoln94@mail.ru (K.B.); dianafuk@yandex.ru (D.F.); mvedunova@yandex.ru (M.V.); suev@mail.ru (E.S.)

**Keywords:** wound coverings, cod collagen, sponge plates, small animals, wound healing, high efficiency

## Abstract

Wound coatings in the form of sponge plates were obtained based on hydrogels of cod collagen (CC) copolymers. The synthesis of CC copolymers with pectin was carried out in the presence of a triethylbor–hexamethylenediamine (TEB-HMDA) complex, which forms free radicals under reaction conditions, and with polyethylene glycol (PEG) during photocatalysis in the presence of RbTe_1.5_W_0.5_O_6_ oxide under visible-light irradiation with a LED lamp. Evaluation of their effectiveness and safety for rapid healing of wounds and burn surfaces has been conducted on small animals (rats). It has shown significantly higher efficiency in comparison with commercial collagen sponges based on bovine collagen. Coatings based on cod collagen contributed to the normalization of microcirculation levels according to the results of laser Doppler flowmetry and a high rate of reduction in the area of the scalped burn wound according to planimetry. The morphological studies indicate complete epithelialization with the formation of scar tissue in all studied groups of animals. The dynamics of microcirculation parameters indicate the repair of thermal burns during local treatment with wound-healing coatings against the background of normalization of the functioning of the microcirculatory system. It is advisable to use new collagen-based polymer sponge plates to increase the effectiveness of wound treatment of various origins, shorten recovery time, and optimize the course of typical physiological reactions during the wound process in order to accelerate tissue regeneration, as well as reduce mortality.

## 1. Introduction

Skin wounds occur as a result of the disruption of skin integrity, which leads to the disruption of their functionality and, in extreme cases, to life-threatening conditions. The development of innovative, multifunctional materials capable of accelerating healing and preventing the development of infection has become a priority in regenerative medicine. Today, scientific publications are often devoted to varying formulations based on natural polymers (proteins and polysaccharides) and biocompatible synthetic polymers (polycaprolactone, polyethylene glycol, etc.), which makes it possible to obtain coatings with new properties [1,2,3,4,5]. It should be noted that collagen is widely studied by scientists all over the world. Thus, the authors of the review [1] consider the structure, preparation and properties of mixtures of collagen with the most common natural polymers, such as chitosan, elastin, etc., and also give examples of new materials based on such mixtures. It is noted that intermolecular interactions between functional groups of biopolymers lead to noticeable changes in the properties of new materials. Collagen- and PEG-based hydrogels demonstrate accelerated functional wound repair [6].

Marine-derived collagen is rapidly becoming the preferred choice due to its unique properties compared to mammalian collagen. It is an optimal natural substrate for such materials for a combination of reasons: it closely resembles the human structure and composition, which means it is biocompatible, a constantly renewable inexpensive resource, does not contradict religious restrictions, does not tolerate animal diseases to humans, etc. [7,8,9,10].

In the development of wound coatings, it is necessary to take into account the problem of antibiotic resistance—the ability of bacteria to survive and multiply despite the presence of antibiotics that previously effectively destroyed these microorganisms [11,12,13]. Therefore, the search for new effective wound coatings with antimicrobial properties is well-justified and deserves the closest attention. Attention is paid to derivatives of various chemical elements. The review [14] summarizes the latest achievements in the field of studying the antibacterial mechanisms of metal nanoparticles and metal oxides (Ag, Au, Cu/CuO, TiO_2_, ZnO, Fe_2_O_3_/Fe_3_O_4_) and discusses approaches to improving the biocidal efficiency of nanoparticles by modifying and creating composites, as well as using light and magnetic fields. In the work in [15], the antibacterial activity of Fe_3_O_4_ was revealed against Gram-positive and Gram-negative bacteria Staphylococcus aureus, Xanthomonas, Escherichia coli and Proteus vulgaris. The studies of antibacterial activity against E. coli and Streptococcus mutans indicate a high biomedical potential of coatings made of TiO_2_ doped with metal ions Ag, Cu, Ga and Zn [16]. The review [17] provides data in which boron-containing compounds show good biocompatibility, which further confirms its potential for biomedical applications. In addition, due to their anti-inflammatory and antimicrobial properties, they can be used in the treatment of various diseases. Wound coatings based on natural polymers with the inclusion of metal nanoparticles and their derivatives are of considerable interest in terms of obtaining materials to accelerate wound healing. The review [18] presents a comprehensive analysis of recent advances in the creation of chitosan materials containing magnetic nanoparticles to accelerate wound healing and discusses potential areas for the development of such research. The strategy of combating bacterial infections by adding metal-based antimicrobials to dressings is promising. Such materials, as noted in the work, have a powerful bactericidal effect, are effective in a wide range and cause minimal resistance. This review examines the various antimicrobial mechanisms of metal-based antimicrobials. It highlights the latest developments in wound dressings containing metal-based antimicrobials, with a focus on the most commonly used metals and types of dressings. Ultimately, the review provides a systematic analysis of the role of metal-based antimicrobials in wound healing, providing valuable information to address the challenges of antibiotic resistance.

The authors of the studies [19,20,21,22,23,24,25,26] have obtained hydrogels of stable three-dimensional structure with a wide pore range from several nm to 70 nm. They used an inert atmosphere, the original initiators in an aqueous dispersion of cod collagen and methyl methacrylate (MMA) with modifiers and biodegradable polymers. Hydrogels release water easily when dried in a vacuum. It is important that they are resistant to mold fungi and bacteria. Cytotoxicity studies of new CC-PMMA-based hydrogels using the methyltetrazolium test (MTT) have identified materials that are promising for creating wound coatings [26,27].

The aim of this work is to develop wound coatings in the form of sponge plates based on hydrogels with biocidal properties and to evaluate their effectiveness and safety for rapid healing of wounds and burn surfaces.

## 2. Materials and Methods

### 2.1. Materials

Commercial reagents were used: acetic acid (analytical grade, Lega, Dzerzhinsk, Russia), sodium hydroxide (pure for analysis, Reahim, Moscow, Russia), pectin (Xlebzernoprodukt, Taganrog, Russia), TEB-HMDA complex (Aviabor, Dzerzhinsk, Russia), acrylic acid (AC) (pure for analysis, Sigma Aldrich, Burlington, MA, USA), triethylene glycol dimethacrylate (TEGDMA) (Himtransit, Dzerzhinsk, Russia) and PEG (Mw = 6000, Norkem, Dzerzhinsk, Russia). The MMA monomer was used (pure for analysis, Sigma Aldrich, Burlington, MA, USA); it was purified from the stabilizer by sequential washing with a solution of sodium hydroxide and cold water until a neutral pH was reached. It was then dried using calcium chloride and distilled in vacuum (1.33 Pa) at a temperature of 40 °C. The RbTe_1.5_W_0.5_O_6_ complex oxide was obtained by the solid-state method, as described earlier [28].

### 2.2. Isolation of Fish Collagen

Collagen was isolated according to the method described in [29] by extraction with acetic acid for one day at room temperature. The resulting acetic acid dispersion with molecular mass 300 kDa was dried to a constant weight under vacuum (1.33 Pa) at 50 °C.

### 2.3. Obtaining Gel for the Sample—“Coating No. 1”

The “Coating 1” sample was synthesized according to a previously described method [25]. A 3% aqueous solution of acetic acid, collagen, and pectin were placed in the reaction flask in a ratio of 59.4:18.4:18.4, heated in a water bath to 60–70 °C, and the TEB-HMDA complex was added at a mass ratio of TEB/natural polymers of 1:5, allowed to stand for 30 min, and then MMA was added (3.6 parts to the total weight reaction mixture). The reaction mixture was kept for another 3 h at 60–70 °C and in an argon atmosphere with constant stirring. A 5% aqueous solution of glutaraldehyde was added to form the hydrogels.

### 2.4. Obtaining Gel for the Sample—“Coating No. 2”

The sample was synthesized according to the previously described method [24]. CC, MMA, TEGDMA, AC, PEG, and water were mixed in a reaction flask (7.7:3.80:0.05:3.80:7.7:76.88 wt.%, respectively), and RbTe_1.5_W_0.5_O_6_ complex oxide was added in the ratio emulsion/catalyst = 180:1. The mixture was bubbled with argon for 15 min, then stirred (600 rpm^−1^) under irradiation with a visible-light-emitting diode lamp (LED, 30 W) in argon current for 5 h. Then, the catalyst was separated by centrifugation. A 5% aqueous solution of glutaraldehyde was added to form the hydrogels.

### 2.5. Production of Sponge Plates

Films were formed on the glass surface by pouring dispersions after synthesis and subsequent drying in a vacuum oven at 50 °C to remove the remaining volatile components. Then, the sponge plates were placed in a container with distilled water. The sponge plates were not sterilized because they have biocidal properties. Sponge plates can be stored in an aqueous solution for 6 months. Sponge plates were applied to the wound without additional sterilization.

### 2.6. Setting up an Experiment with Animals

The research was conducted in accordance with the Helsinki Declaration (2000). The animal experiment was conducted in compliance with the principles of humanity, in accordance with the directives of the European Community (No.86/609/EEC, Strasbourg, 1986).

The experiment was conducted on 40 male Wistar rats weighing 250–300 g. The animals were kept in standard vivarium conditions in cages with free access to food and water on a diet, according to the standards “Maintenance of experimental animals in research Institute nurseries” [30].

Before the experiment, the animals were quarantined for 14 days on standard food and water rations in natural light and air temperature in the range of 18–22 °C, in accordance with the “Sanitary rules for the establishment, equipment and maintenance of experimental biological clinics.” At the end of the quarantine, a general assessment of the physiological state of the rats was carried out by the type of mucous membranes, the level of motor activity, and the consumption of feed and water. After 14 days of adaptation to the conditions of the local vivarium and quarantine, the animals were randomized into four equal groups using a randomization method—a table of random numbers. Thermal skin burns were applied to rats of all groups.

Grade II thermal contact injury with an area of 10% of the body surface was applied by contact using a steel stencil (incandescent temperature 240 °C, exposure 5 s) to a pre-epilated area of the back skin under general anesthesia (Zoletil 100 (60 mg/kg) (Virbac SA, Carros, France) + XylaVET (6 mg/kg) (ZylaVet, Saint-Petersburg, Russia)). After 3 h, all animals, regardless of group membership, also underwent necrectomy of the burn scab under general anesthesia: using a scalpel, a vertical fringing incision was made along the border of the burn wound to the intradermal muscle layer (panniculus carnosus), and the skin area was resected by acute dissection and detachment of the dermis from the intradermal muscle layer [31].

**Figure 1:**
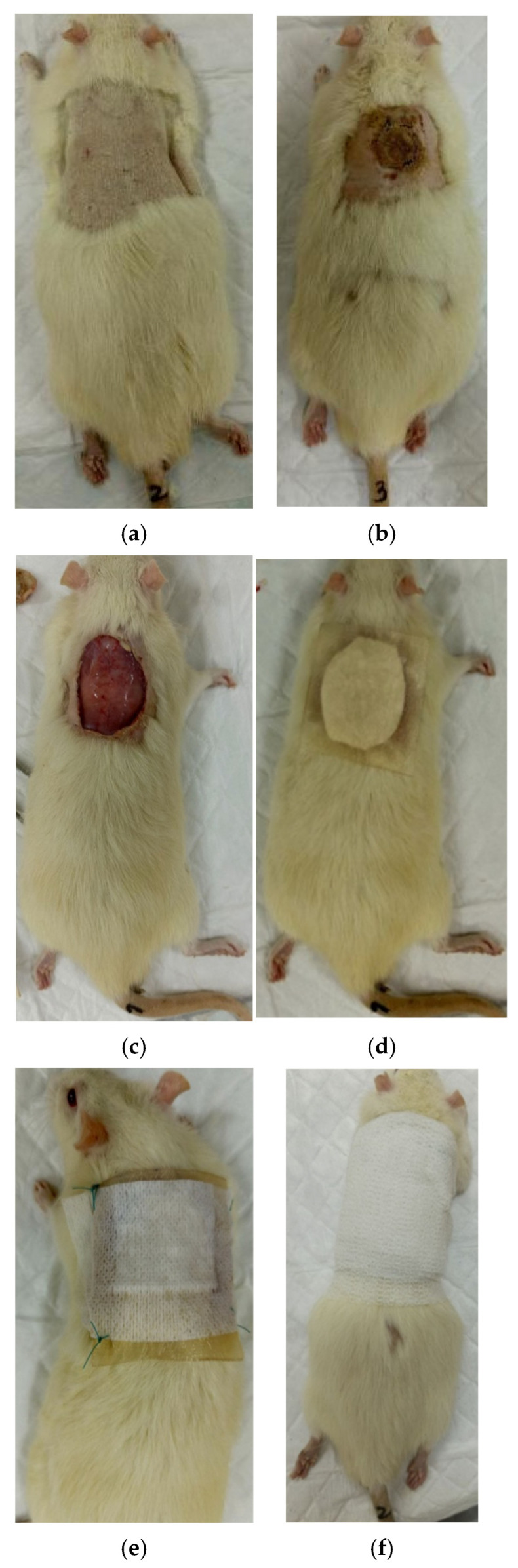
Stages of burn wound application and coating: (**a**) before the burn, (**b**) after the burn (day 0), (**c**) necrectomy after the burn (day 0), (**d**) coating and hydrogel dressing, (**e**) Cosmopor E dressing, (**f**) coating with Peha-haft self-locking bandages.

Wound management was performed in a closed manner under sterile hydrogel dressings and Cosmopor E (Hartmann, Heidenheim an der Brenz, Germany), as well as Peha-haft self-fixing bandages (Hartmann, Germany). The test coating was placed between the wound and the hydrogel dressing. Wounds and bandages were wetted daily with a 4% gentamicin solution (Dalkhimpharm, Khabarovsk, Russia). Bandages were applied once every 3–4 days under general anesthesia (Zoletil 100 (60 mg/kg) + XylaVET (6 mg/kg)) (Figure 1). The animals were euthanized on day 28 under anesthesia (Zoletil 100 (60 mg/kg) + XylaVET (6 mg/kg)), intermediate studies were performed on days 7, 14, and 21.

Group 1 (control 1) comprised rats with a spontaneous course of the wound process without treatment, and no local applications were performed. To the rats of the control group 2 (control 2), a commercial collagen wound coating was applied to the wound surface (Zelenaya Dubrava, Dmitrov, Russia). Rats of group 3—experiment 1—were coated with coating No.1 on the affected area. Group 4 rats—experiment 2—were coated with coating No.2 on the affected area.

Microcirculation parameters were evaluated in 10 intact rats kept in conditions similar to the animals included in the experiment, but without any interventions. By the time of the study, all the animals were healthy, with no changes in appetite, behavior, sleep patterns, or wakefulness. Microcirculation and burn area were assessed on the day of the simulation and once every 7 days, up to the 28 days of the experiment.

### 2.7. Research Methods

#### 2.7.1. Sponge-Plate Analysis

The analysis of dry sponge plates to determine the collagen content was carried out by elemental analysis (CHNS) on a vario EL cube element analyzer for simultaneous determination of CHNS(O).

The molecular weight characteristics of the aqueous phases of synthesis after coagulation were determined using gel-penetrating chromatography (GPC). Aqueous solutions were analyzed using a high-performance liquid chromatograph, manufactured by Shimadzu CTO 20A/20A C (Kyoto, Japan). Separation was performed using a Tosoh Bioscience TSKgel g3000swxl column with a pore diameter of 5 microns and a low-temperature light-scattering detector (ELSD-LT II). The eluent consisted of 0.5 M acetic acid solution, the flow rate was 0.8 mL/min, and narrowly dispersed dextran standards with a molecular weight range (MW) of 1–410 kDa (Fluca, Buchs, Switzerland) were used for calibration.

#### 2.7.2. Planimetric Assessment of the Process of Repair of Burn Wounds in Rats

**Figure 2:**
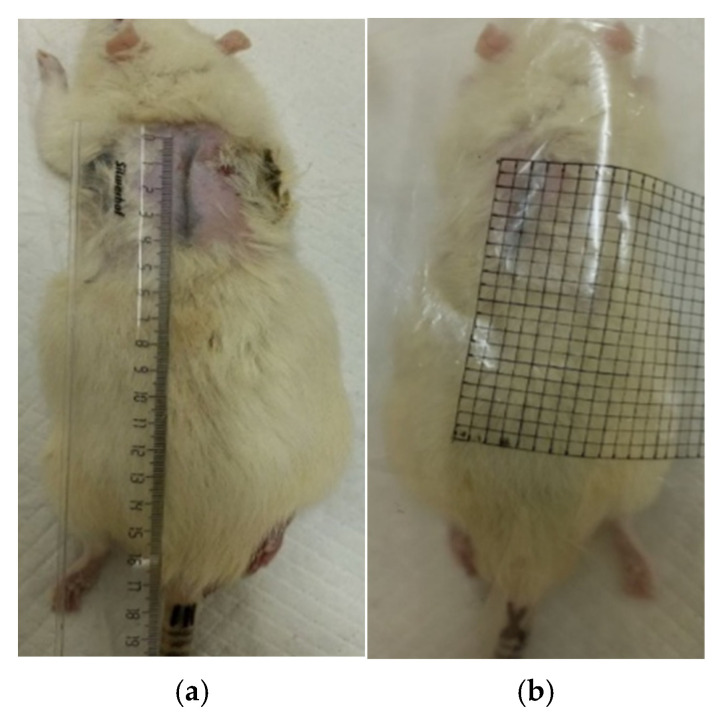
Planimetry of wounds: (**a**) scar length; (**b**) millimeter paper.

**Figure 3:**
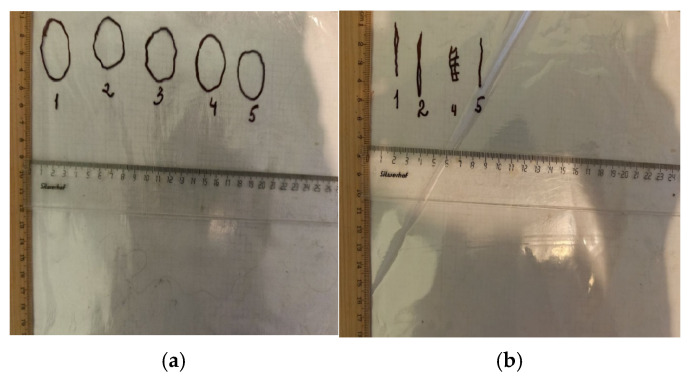
Contours of defects: (**a**) Experiment No.3 (day 1), (**b**) Experiment No.3 (day 28).

The dynamics of the burn defect healing were assessed using the planimetric method (Figure 2). Starting from the second day after the injury, the contours of each defect were transferred daily to a transparent film (Figure 3), then its area was calculated using graph paper. The absolute area (S, cm^2^) of the superficial skin defect was calculated using Formula (1) [32]:

S = n + ½ k,(1)

where n is the number of 1 × 1 mm squares completely located within the contour of the wound; k is the number of 1 × 1 mm squares partially located within the contour of the wound.

The relative decrease in wound area ∆S per day, characterizing the rate of burn defects healing, was calculated by Formula (2):

∆S = (S_2_ − S_1_)/S_2_ × 100%, (2)

where S_2_—initial burn area, S_1_—the area of the wound on the current measurement day.

#### 2.7.3. Morphological Studies of the Skin

The skin pieces were fixed in a 10% formalin solution. Serial sections of 4–6 microns thick material from paraffin blocks (KhimPEC, Moscow, Russia) were made on an electronic microtome MSE (Inmedprom, Moscow, Russia). The sections were stained with hematoxylin (HiMedia Laboratories, Mumbai, India) and eosin Y (Panreac-AppliChem, An ITW Company, Barcelona, Spain) [33]. Microscopy and photo documentation were performed using the Leica DM 1000 morphometric complex (Leica Microsystems, Wetzlar, Germany) and a digital camera Leica DFC 290 HD (Leica Microsystems, Wetzlar, Germany).

#### 2.7.4. Microcirculation Research

The microcirculation level in intact skin and burn wound was assessed by laser Doppler flowmetry (LDF) [34,35,36] using a LAKK-M laser analyzer (version 2) (Lazma, Moscow, Russia), recording for 3 min.

When examining the level of tissue microhemocirculation, the microcirculation index (MI) was calculated using the formula:

MI = K ∙ N_er_ ∙ V_av_, (3)

where K—proportionality coefficient (K = 1); N_er_—the number of erythrocytes; V_av_—the average rate of erythrocytes in the probed volume.

MI characterizes the average perfusion level (the average flow of erythrocytes) per unit volume of tissue per unit time. MI is measured in perfusion units. This indicator provides an integral assessment of the microcirculation state of the tissue area under study.

Using the LDF 3 program, a wavelet analysis was performed to calculate the frequency fluctuations in blood flow in order to identify the role of active (endothelial fluctuations (E)—0.01–0.08 Hz, neurogenic fluctuations (N)—0.08–0.2 Hz, myogenic fluctuations (M)—0.2–0.7 Hz) and passive (respiratory (R)—0.7–2 Hz, cardiac (C)—2–5 Hz) factors regulating microcirculation, followed by calculation of the bypass index (BI).

### 2.8. Statistical Processing of Results

Statistical processing of the obtained data was carried out using the Microsoft Excel software package, Statistica 6.0 (Statsoft Inc., Tulsa, OK, USA). The results of the experimental study are presented in the form of an arithmetic mean and a standard deviation (M ± σ). Before conducting intergroup comparisons of the data, the nature of their distribution was assessed using the Shapiro–Wilk criterion. The reliability of the intergroup differences in the results was assessed using Student’s *t*-test with Bonferroni correction for multiple comparisons. The differences were considered statistically significant when *p* < 0.05.

## 3. Results

### 3.1. Sponge Plates for Wound Coverings Based on Fish Collagen

**Figure 4:**
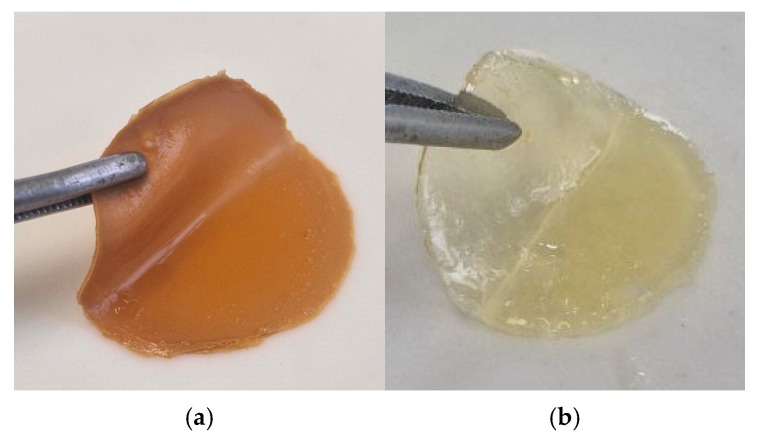
Photos of sponge plates: (**a)** Coating No.1, (**b**) Coating No.2.

Sponge plates based on hydrogels with biocidal properties were obtained on the basis of two developments. The “Coating No.1” sample was synthesized according to the previously described method [25] based on collagen–pectin–methyl methacrylate copolymers obtained in the presence of triethylborane. The “Coating No.2” sample was obtained as a result of the hydrogel formation from the components CC-MMA-TEGDMA-AC-PEG-water in dispersion with a photocatalyst RbTe_1.5_W_0.5_O_6_ under visible-light LED lamp (LED, 30 W) irradiation [24]. The biocidal properties of these materials have been established in previous studies [26,27]. Sponge plates in the shape of a circle are shown in Figure 4.

It was found that the nitrogen content in the dry samples “Coating No.1” and “Coating No.2” according to the elemental analysis are ~5–6% and ~6–7%, respectively. The collagen content in the obtained coatings was calculated taking into account the fact that the nitrogen content in pure collagen is 16–18% (100% protein without synthetic fragments), it was 28–38% and ~33–44%, respectively. In the aqueous phase after coagulation of the hydrogel, the following high-molecular-weight fragments are identified:

- for the “Coating No.1” sample, small amounts (at the level of 1% of the total mass of the starting substances) were not integrated into the matrix of the grafted copolymer PMMA-collagen, PMMA-pectin;
- for the “Coating No.2” sample, small amounts (at the level of 1% of the total mass of the starting substances) were not integrated into the matrix of the grafted copolymer PMMA-collagen and PEG;
- for both samples, insignificant amounts of low-molecular-weight collagen with MW ~10 kDa and ~20 kDa.

To test the effectiveness and safety of coatings for rapid healing of wounds and burn surfaces, samples were made in the form of sponge plates measuring 3 cm × 3 cm in distilled water.

### 3.2. The Results of a Planimetric Assessment of the Repair Process of Burn Wounds in Rats During Their Treatment with Collagen Preparations

After simulating the burn during the day, the activity of the animals in all groups was reduced; they ate small portions. Motor activity and appetite were fully restored in the rats of the experimental groups and control group 2 on the third day after applying the thermal burn; however, signs of physiological discomfort were observed in the animals of control group 1.

**Figure 5:**
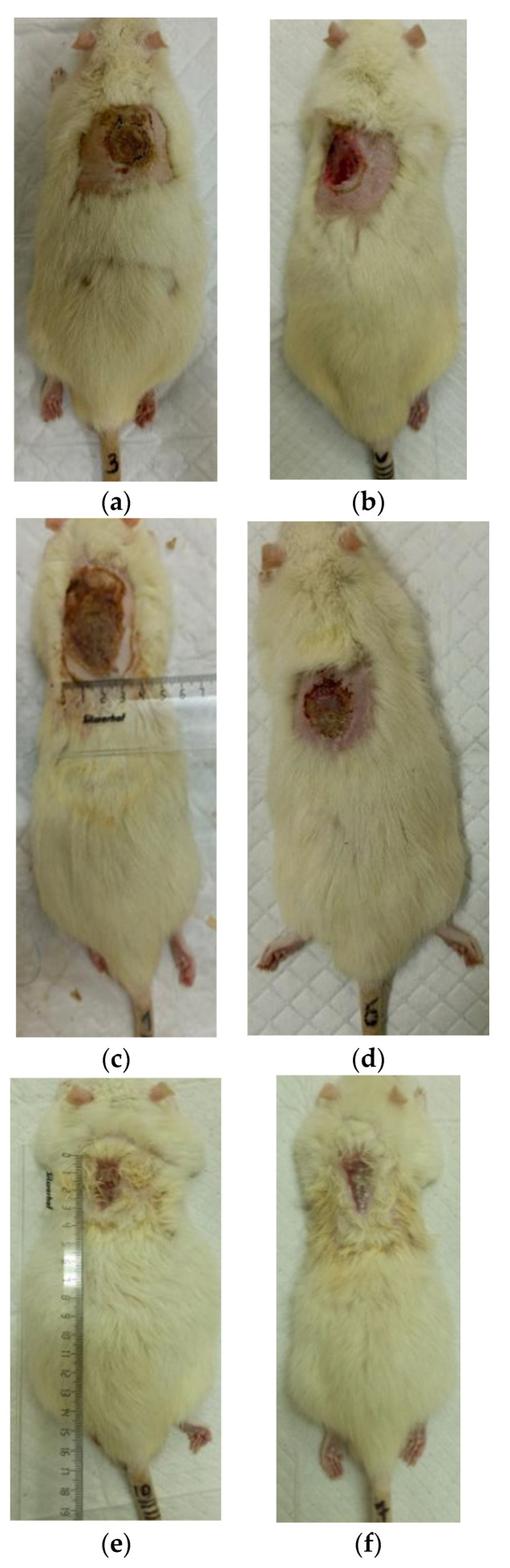
Regeneration of the skin in control 1 (burn without treatment): (**a**) after the burn (day 0), (**b**) scalped wound (day 0), (**c**) day 7 after the burn, (**d**) day 14 after the burn, (**e**) day 21 after the burn, (**f**) day 28 after the burn.

**Figure 6:**
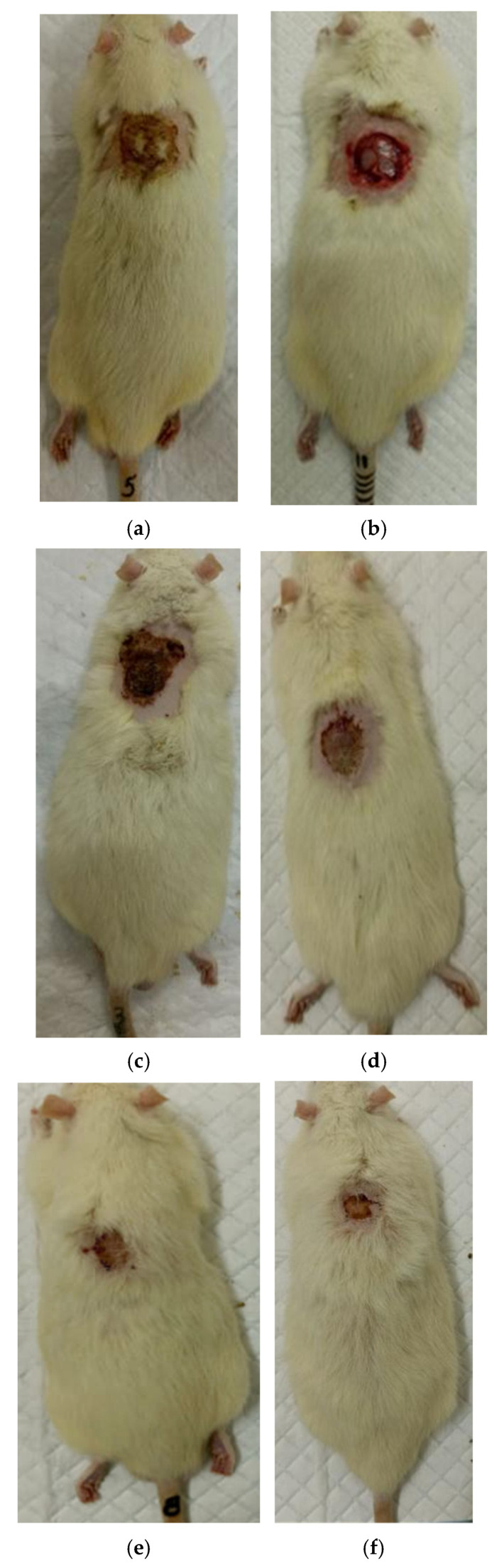
Regeneration of the skin in control 2 (commercial collagen wound coating): (**a**) after the burn (day 0), (**b**) scalped wound (day 0), (**c**) day 7 after the burn, (**d**) day 14 after the burn, (**e**) day 21 after the burn, (**f**) day 28 after the burn.

**Figure 7:**
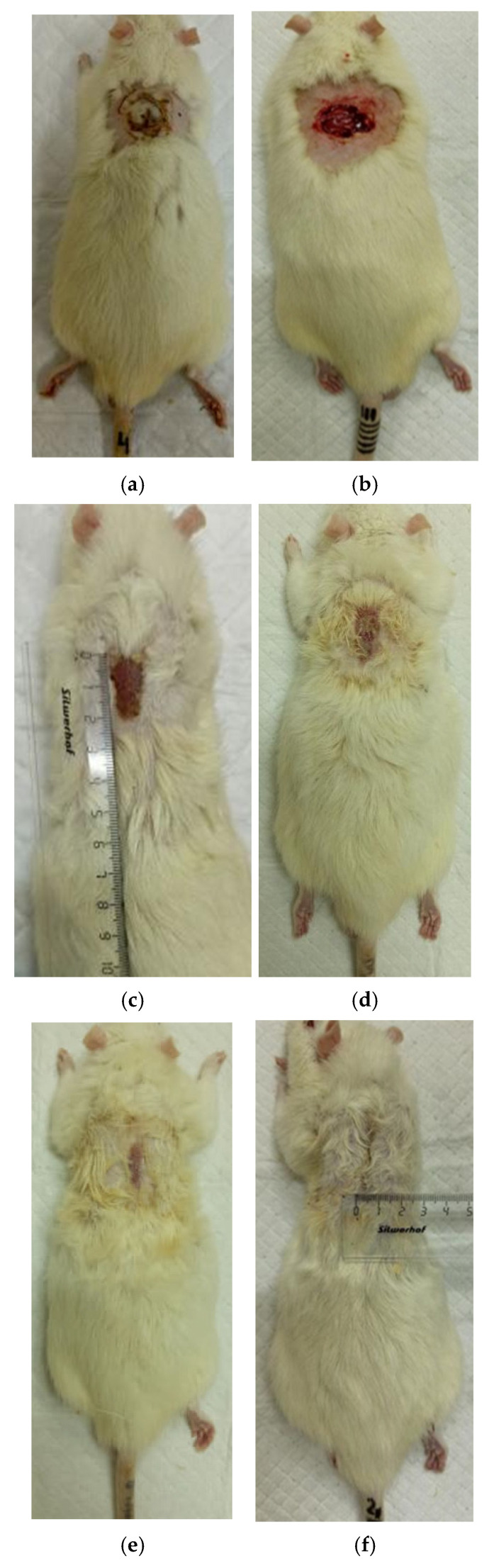
Regeneration of skin in experience 1 (coating No.1): (**a**) after a burn (day 0), (**b**) scalped wound (day 0), (**c**) day 7 after a burn, (**d**) day 14 after a burn, (**e**) day 21 after a burn, (**f**) 28 days after the burn.

**Figure 8:**
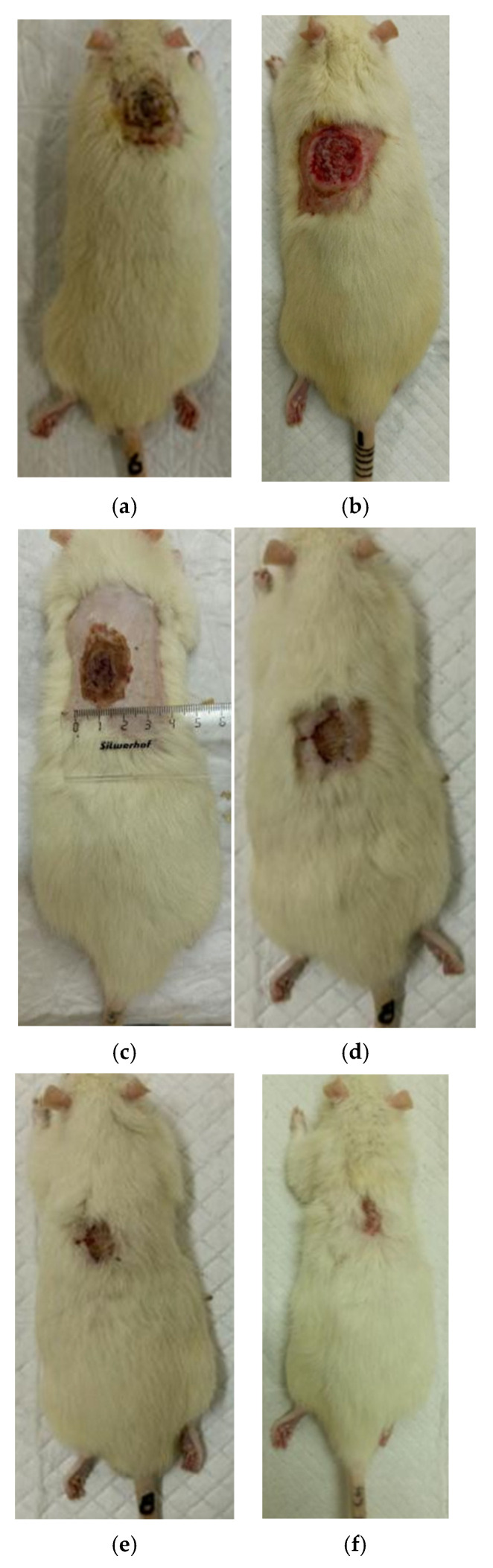
Regeneration of skin in experience 2 (coating No.2): (**a**) after a burn (day 0), (**b**) scalped wound (day 0), (**c**) day 7 after a burn, (**d**) day 14 after a burn, (**e**) day 21 after a burn, (**f**) 28 days after the burn.

The process of burn wound repair in rats during their treatment with collagen preparations is shown in Figure 5, Figure 6, Figure 7 and Figure 8. A planimetric assessment of the thermal skin lesions area on the seventh day of the experiment showed that in animals of the first experimental group, the absolute values of this parameter were significantly lower in comparison with both control groups (Table 1).

At the same time, the rate of scab area reduction on day 7 was 18.4% in the first experimental group and 10.1% in the second experimental group versus 4.3% and 4.3% in the control groups 1 and 2, respectively. The recorded higher rates of recovery processes in the experimental groups of rats at this stage of regeneration, from the standpoint of morphological changes, are based on rapid relief of inflammation and the absence of a tendency to spread inflammatory infiltrate into surrounding tissues. It characterizes the process of cleansing the wound field as better and more intense and leads to a change in the stage of inflammation to the next one—proliferation with the formation of granulation tissue in the area of the injury.

On the 14th day of the experiment, the areas of thermal skin defects continued to decrease at different rates in animals of the compared groups. So, in rats of the first experimental group with coating No.1, it was 62.6%; in the second experimental group with coating, No.2—53.2%; and while in control groups 1 and 2, it was 44.0% and 48.9%, respectively. At the same time, the wound area index in absolute terms was statistically significantly lower only in rats of the first experimental group compared to control group 2 (Table 1). It is obvious that the processes of wound process proliferation under the influence of coating No.1 proceeded more successfully. The granulation tissue was actively formed, which is a substrate for the growth of regenerating epithelium. Macroscopically, it led to a rapid reduction in the area of the skin defects. Judging by the rate of decrease in the area of thermal skin defects in animals of both control series, these processes were not so active.

On the 21st day of the experiment, the areas of thermal skin defects also continued to decrease at different rates in animals of the compared groups. So, in rats of the first experimental group with coating No.1, it was 87.7%; in the second experimental group—85.1%; in control groups 1 and 2—80.7% and 82.4%, respectively. In rats of all experimental groups, the wound area index in absolute terms was statistically significantly lower compared to the control group 2.

On the 28th day of the experiment, the burn zone in the rats of the experimental groups was represented by connective tissue scars and in places resembled intact skin; the coat began to recover (Figure 7f and Figure 8f), which indicates the near-completion of regenerative processes under the influence of the studied coatings.

In the rats of both control groups, burn regeneration has not yet been completed on day 28. So, in control group 1, on day 28, the area of the residual burn defect still had a value of 2.76 ± 0.03 cm^2^, which was 12.3% of the initial one at the beginning of the experiment. In control group 2, by the time the animals were removed from the experiment (day 28), the residual defect area was 1.31 ± 0.02 cm^2^, or 6.0% of the initial one.

### 3.3. Results of Morphological Examination

**Figure 9:**
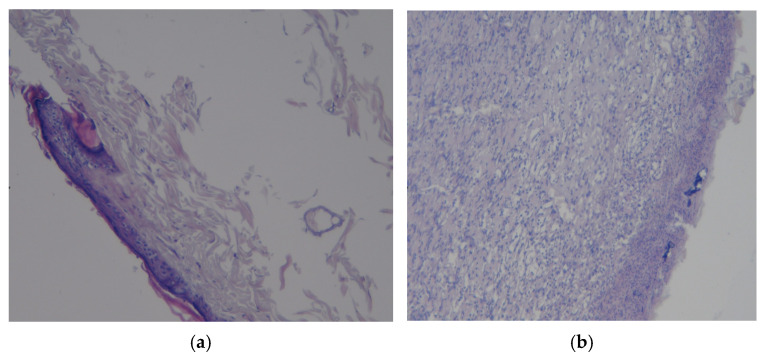
Preparations of skin flap sections from control 2: (**a**) magnification ×10; (**b**) magnification ×20.

In the presented preparation (Figure 9a) of a commercially coated rat skin flap, the signs of inflammation are minimally pronounced and are represented by single-tissue basophils and leukocytes. There is no ulcerative defect, though a large number of sebaceous glands are noted. No signs of bacterial infection or neoplasia were found. The revealed changes in the tissues may conditionally correspond to the norm.

The histological picture of the preparation of a commercially coated rat skin flap is shown in Figure 9b. It shows the mixed-cell inflammation, which is moderately pronounced in the dermis, an ulcerative defect with the formation of a “crust” at the surface and no signs of bacterial infection or neoplasia. The revealed changes in the tissues correspond to regenerative changes with epithelialization and scar tissue formation.

**Figure 10:**
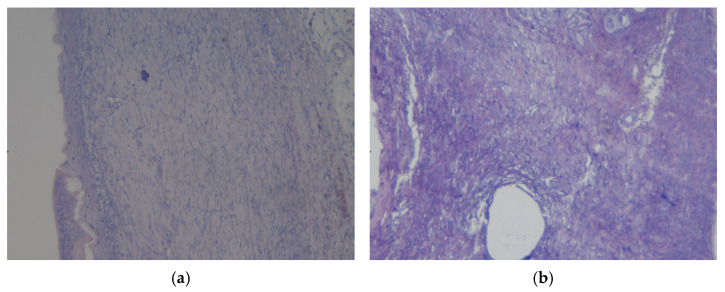
Preparations of skin flap sections from experience 1 (coating No.1): (**a**) magnification ×10; (**b**) magnification ×20.

On the preparation of a rat skin flap cut with experimental coating No.1 (Figure 10a), a mixed-cell inflammation in the dermis was noted in the histological picture, but no signs of bacterial infection or neoplasia were detected. Thus, the revealed changes in the tissues correspond to regenerative changes with epithelialization and scar tissue formation.

The histological picture of the rat skin flap preparation with experimental coating No.1 (Figure 10b) was characterized by changes macroscopically corresponding to the formation of vesicles or bullae in the epidermis; mixed-cell inflammation in the dermis was noted, and tissue basophils were observed at the level of the deep dermis. The upper layers of the dermis are loosened and swollen. Consequently, bullous skin changes with the formation of scar tissue have been identified.

**Figure 11:**
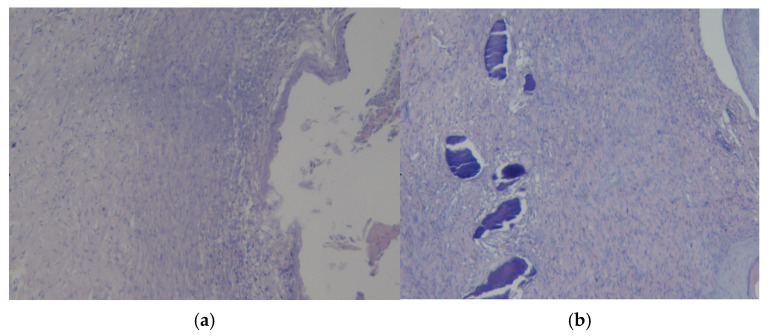
Preparations of skin flap sections from experience 2 (coating No.2): (**a**) magnification ×10; (**b**) magnification ×20.

In a rat with a burn and experimental coating No.2 (Figure 11a), the histological picture of the skin flap preparation is characterized by mixed-cell inflammation in the dermis. A large ulcerative defect with the formation of a “crust” is observed near the surface, and foci of caseous or “dry” necrosis are present in the underlying scar tissue. There were no signs of bacterial infection or neoplasia. Thus, the revealed changes in the tissues correspond to regenerative changes with epithelialization and scar tissue formation.

Figure 11b also shows a histological picture of the preparation of a rat skin flap cut with experimental coating No.2. Mixed-cell inflammation is present in the dermis. A large ulcerative defect with the formation of a “crust” is observed near the surface. There are pockets of caseous or “dry” necrosis in the underlying scar tissue. There were no signs of bacterial infection or neoplasia. Consequently, the revealed changes in the tissues also correspond to regenerative changes with epithelialization and scar tissue formation.

### 3.4. Assessment of Microcirculation in the Healing Process of Burn Wounds

After thermal injury, the negative dynamics of microcirculation changes were revealed at the local level. The volume of blood flow decreased: on day 0 after the burn, the microcirculation index decreased in all the studied groups by 47–52% compared with the index of intact rats.

The compensatory response from regulatory influences during the burn was an increase in the factors activity of active and passive regulation of the microcirculation system (an increase in the activity of endothelial, neurogenic and myogenic influences, as well as an increase in the influence of passive regulatory factors—respiratory and pulse waves) relative to the values of intact animals. At the same time, the bypass rate immediately after injury was less than 1.0, which was partly supposed to compensate for the lack of arterial blood flow; however, the BI decreased compared to the intact rats rate in all groups; therefore, the volume microcirculation in the periphery was sharply reduced and did not fulfill its transport function (Table 2).

The results showed that the use of wound-healing bandages No.1 and No.2, as well as commercial collagen wound coating for burns caused normalization of microcirculation and regulatory factors of microcirculation regulation in all study groups by day 28 (Table 2).

## 4. Discussion

Thus, laboratory methods have been developed for the production of wound coatings in the form of sponge plates based on a copolymer of cod collagen and MMA with the inclusion of pectin, polyethylene glycol and crosslinking organic components in the composition of the material. Tests of wound healing on small animals (rats) using new coatings have shown significantly higher efficiency compared to commercial samples based on bovine collagen. The use of coatings based on cod collagen contributed to the normalization of microcirculation levels according to the laser Doppler flowmetry results, as well as a high rate of area reduction in the scalped burn wound according to planimetry, which may be associated with an improvement in trophic processes in the skin dermal layers. The presence of statistically significant differences in the burn defects areas between control groups 1 and 2 only from day 21 indicates that the commercial drug does not have a rapidly pronounced stimulating effect. At the same time, the reparative potential of experimental coatings No.1 and No.2 is already topically realized at the stages of inflammation and proliferation. The pro-regenerative properties of the studied coatings manifested themselves in a significant accelerated healing of experimental thermal skin burns. Both coatings showed a high regenerative potential; however, when using coating No.1, healing of the affected area was faster, and when using coating No.2, an earlier restoration of microcirculation was detected, which may be due to a number of reasons. It can be suggested that, firstly, as noted in the introduction [1,2,3,4,5], there is often an increase in the effectiveness of mixtures of collagen with biocompatible polymers in comparison with collagen coatings. In addition to collagen, pectin is used in coating No.1, and PEG is used in coating No.2. This may be the reason for the different courses of wound healing. Secondly, nanoparticles of the boron-containing initiator in the first coating and RbTe_1.5_W_0.5_O_6_ oxide in the second, as well as other already-known metal and oxide nanoparticles [14,15,16,17,18], can accelerate wound healing, and also in various ways.

The morphological studies indicate complete epithelialization with the scar tissue formation in all groups of animals studied. The study of the dynamics of microcirculation indicators shows that the repair of thermal burns during local treatment with wound-healing coatings proceeds against the background of normalization of the functioning normalization of the microcirculatory system. The stimulating reparative effect of the experimental coatings is associated with a statistically significant decrease in the regulatory microcirculation components at all stages of the experiment.

## 5. Conclusions

Studies of collagen-based polymer materials based on a thermal burn model can be used to develop a treatment strategy for not only burns but also trophic ulcers, and can also be used in extreme military medicine, rehabilitation, and sports medicine. Thus, it is advisable to use the studied collagen-based polymer materials to increase the effectiveness of wound treatment of various origins, namely, to shorten the recovery time and to optimize the course of typical physiological reactions during the wound process in order to accelerate tissue regeneration, as well as reduce mortality.

## Figures and Tables

**Table 1:** Dynamics of the skin defects area (S, cm^2^) in rats during thermal burn regeneration.

Group	0 Day	7 Days	14 Days	21 Days	28 Days
Control 1 (without treatment)	22.52 ± 0.71	21.55 ± 0.28	12.62 ± 0.15	4.34 ± 0.11	2.76 ± 0.03
Control 2 (commercial coating)	21.81 ± 0.53	20.93 ± 0.47	11.15 ± 0.09	3.83 ± 0.02 *	1.31 ± 0.02 *
Experience 1 (Coating No.1)	21.96 ± 1.02	17.91 ± 0.52 */∆	8.21 ± 0.12 */∆	2.70 ± 0.04 */∆	0 (scar) */∆
Experience 2 (Coating No.2)	22.30 ± 0.72	20.04 ± 0.67	10.43 ± 0.38 *	3.32 ± 0.05 */∆	0.68 ± 0.01 */∆

Note: *—the difference is statistically significant (*p* < 0.05) between the parameter in animals of the experimental group and the control group 1; ∆—the difference is statistically significant (*p* < 0.05) between the parameter in animals of the experimental group and the control group 2.

**Table 2:** Microcirculation status in thermal injury when using new materials to close wound surfaces.

Group	MI, perf.un.	E, RVU	N, RVU	M, RVU	R, RVU	C, RVU	BI, perf.un.
Intact rats	9.45 ± 0.85	12.93 ± 1.15	9.15 ± 0.86	8.37 ± 0.81	5.80 ± 0.49	3.33 ± 0.27	1.14 ± 0.08
Control 1, 0 days	4.86 ± 0.52 *	14.73 ± 1.28	11.02 ± 0.93	11.74 ± 0.77 *	8.26 ± 0.52 *	4.99 ± 0.28 *	0.92 ± 0.03 *
Control 1, 28 days	6.99 ± 0.51 *	13.07 ± 1.12	10.29 ± 1.13	10.04 ± 1.36	7.48 ± 0.65 *	4.12 ± 0.36	0.98 ± 0.01
Control 2, 0 days	5.01 ± 0.39 *	13.81 ± 1.05	10.87 ± 0.93	12.02 ± 1.34 *	7.96 ± 0.83 *	5.04 ± 0.20 *	0.92 ± 0.05 *
Control 2, 28 days	7.91 ± 0.46	12.85 ± 1.03	10.03 ± 0.42	9.83 ± 0.56	6.27 ± 0.16	3.85 ± 0.22	1.03 ± 0.02
Experience 1, 0 days	4.95 ± 0.37 *	14.56 ± 1.23	10.92 ± 0.65	12.13 ± 0.83 *	8.51 ± 0.47 *	4.73 ± 0.16 *	0.91 ± 0.03 *
Experience 1, 28 days	8.76 ± 0.53 Δ	14.02 ± 1.11	9.48 ± 0.37	9.24 ± 0.61	6.35 ± 0.42	3.50 ± 0.17	0.98 ± 0.07
Experience 2, 0 days	4.53 ± 0.48 *	13.59 ± 1.04	11.65 ± 1.23	10.84 ± 1.32	8.64 ± 0.33 *	5.12 ± 0.11 *	0.94 ± 0.02 *
Experience 2, 28 days	8.44 ± 0.61 Δ	13.76 ± 1.27	10.48 ± 0.72	9.23 ± 0.36	6.12 ± 0.30	3.88 ± 0.29	1.11 ± 0.03

Note: *—the differences are statistically significant compared to intact rats (*p* < 0.05); Δ—the differences are statistically significant compared to the control 1 (*p* < 0.05).

## Data Availability

The original contributions presented in this study are included in the article. Further inquiries can be directed to the corresponding author.
